# Virtual and clinical implant placement after ridge preservation in periodontally compromised molars: retrospective study

**DOI:** 10.1186/s12903-026-07921-7

**Published:** 2026-02-18

**Authors:** Haoyun Zhang, Yiping Wei, Wenjie Hu, Tao Xu, Min Zhen, Liping Zhao, Cui Wang, Ziyao Han, Ning Wei, Kwok-Hung Chung

**Affiliations:** 1 Department of Periodontology, Peking University School and Hospital of Stomatology & National Center of Stomatology & National Clinical Research Center for Oral Diseases & National Engineering Research Center of Oral Biomaterials and Digital Medical Devices., No.22, Zhongguancun South Avenue, Haidian District, Beijing, 100081 China; 2 Department of Emergency, Peking University School and Hospital of Stomatology & National Center of Stomatology & National Clinical Research Center for Oral Diseases & National Engineering Research Center of Oral Biomaterials and Digital Medical Devices, Beijing, China; 3 Department of Restorative Dentistry, School of Dentistry, University of Washington, Seattle, WA USA

**Keywords:** Ridge preservation, Molar extraction, Severe periodontitis, Dental implant, Cone beam computed tomography, Virtual implant placement

## Abstract

**Aims:**

The purpose of this study is to assess the possibility and needs for bone augmentation in virtual planning and clinical treatment of prosthetically guided dental implants (PGI) following alveolar ridge preservation (ARP) in periodontally compromised molar extraction sites and to analyze the influencing factors.

**Materials and methods:**

One hundred forty three subjects with 191 molars were included. Radiographic data assessed baseline ridge height and width in variable sites. Virtual implant planning with 4.1/4.8 mm diameter, 8/10 mm length implants was performed using CBCT. The feasibility of simple implant placement and the need for augmentation during implant therapy were assessed. According to the medical records, the implant therapy modalities for sites that received dental implant treatment were also counted.

**Results:**

Results of virtual implant planning showed that a few sites (6.81%) required staged augmentation for 4.1 × 8 mm and 4.8 × 8 mm implants, while 59.16% and 45.55% of the sites were possible for simple implant placement, respectively. For all cases clinically treated with implant therapy, 76.1% received simple implant placement, and 3.15% received staged augmentation. Implant treatment modalities were most influenced by location (maxillary/mandible) and the baseline buccal and lingual/palatal bone defects.

**Conclusions:**

PGI placement is possible in periodontally compromised molars following ARP, and only a few sites require complicated augmentation procedures. Location and baseline buccal and lingual/palatal bone defects are the influencing factors for implant treatment planning.

**Supplementary Information:**

The online version contains supplementary material available at 10.1186/s12903-026-07921-7.

## Introduction

Advanced periodontitis was the sixth most prevalent health condition, affecting approximately 10.8% of the population worldwide [1]. The total burden of this disease has increased in recent decades [2], and it is now the leading cause of tooth loss in adults [3, 4]. Molars are instrumental in chewing function, but their anatomy and position in the mouth make them challenging for periodontal treatment and maintenance [5, 6]. According to the available research, molars have the highest risk of tooth loss following periodontal therapy [7].

A systematic review analyzed the changes in alveolar ridge dimensions after extraction following unassisted socket healing and showed variable resorption and more reduction in molar sites [8]. Tooth loss due to advanced periodontitis often leads to severe damage to the alveolar ridge. Researches support that ongoing ridge dimension loss appears after natural healing. These ridge deficiencies may affect subsequent implant procedures [9, 10].

For clinicians, reducing horizontal and vertical alveolar bone resorption and maintaining a good alveolar bone contour after tooth extraction is an important challenge. Many previous studies have proposed solutions such as less traumatic extraction procedures [11], immediate implant placement combined with prosthodontic methods [12], and alveolar ridge preservation (ARP) using various biomaterials, including socket filling materials like xenografts, allografts, alloplasts [13] and collagen [14], as well as socket sealing materials like barrier membranes, collagen sponge, and soft tissue grafts [15]. The goals of ARP are to limit dimensional changes in the alveolar ridge after extraction, facilitating implant placement without additional extensive bone and soft tissue augmentation procedures, to promote new bone formation in the healing alveolus, and to promote soft tissue healing at the entrance of the alveolus and preserve the alveolar ridge contour [16, 17]. Some studies demonstrated the safety and effectiveness of this ridge preservation technique in periodontally compromised sockets [18]. Since then, a growing number of investigations have reported the outcomes of ARP in these sites and concluded that ARP effectively reduces bone resorption [19–21].

In previous studies, evaluation of ARP effectiveness primarily focused on vertical and horizontal changes in alveolar ridge dimensions [21–24]. These results provide valuable information on the efficacy of ARP. The ideal implant position with the prosthetic axis is one of the critical parameters for implant function, esthetics, and long-term maintenance [25]. Thus, the feasibility of prosthetically guided implant placement must also be evaluated. Although some studies have investigated the treatment modalities for implant placement following ARP, the existence of deviation in implant surgery, different implant options, and variable criteria for augmentation are objective [26–29]. Fok et al. performed virtual implant placement, which is an available and clinically relevant method to assess the outcomes of implant placement at sites with first molar loss due to terminal periodontitis [30]. However, there is limited literature about the effectiveness of ARP procedures on periodontally compromised molar extraction sites in terms of ideal implant placement.

The dimensional changes of alveolar ridge after ARP are related to implant treatment. Systemic influencing factors on alveolar ridge changes include age, systemic diseases, smoking, using medications affecting bone metabolism, and radiotherapy exposure [31–33], among others. Previous studies preliminarily explored the impact of anatomical factors of extraction socket on ARP effectiveness, such as tooth type, jaw location, severity of bone defect, and whether adjacent teeth were extracted [21, 24, 34–36]. However, research on factors influencing implant outcomes after ARP remains limited.

The present retrospective study aims to assess the possibility and need for bone augmentation in virtual implant planning and clinical treatment of prosthetically guided dental implants (PGI) following ARP at periodontally compromised molar extraction sites. Implant success depends on biological and mechanical factors. Overloading can cause bone resorption around implants. Increasing bone-implant contact can reduce this risk by improving osseointegration and stress distribution [37, 38]. Therefore, this study evaluated conventional implants with diameters of 4.1/4.8 mm and lengths of 8/10 mm, instead of using narrow or short implants to reduce the need for bone augmentation during implant placement. The primary objective is to evaluate the outcomes of virtual implant planning and clinical implant therapy. The secondary aim is to analyze the factors influencing virtual implant placement.

## Material and methods

### Study design and setting

This retrospective study was approved by the Ethics Committee of Peking University School and Hospital of Stomatology (PKUSSIRB-2024101127) and was performed strictly with the World Medical Association Declaration of Helsinki. This study is based on anonymized data collected routinely, and the design and report of this study followed the Strengthening the Reporting of Observational Studies in Epidemiology (STROBE) guidelines for observational studies.

### Participants

A retrospective chart review was conducted to identify patients treated with alveolar ridge preservation (ARP) following tooth extraction at Peking University School and Hospital of Stomatology from January 2015 to July 2021, performed by two periodontists (WH and TX).

The records of patients who met the following inclusion criteria were selected: a) over 25 years old at the time of surgery; b) provided written informed consent for the surgery and using clinical and radiographic records for research purposes; c) had hopeless molars as a consequence of severe periodontitis with or without endodontic lesion (probing depth ≥ 6 mm, clinical attachment loss ≥ 5 mm, mobility > II degree) [39, 40]; d) underwent minimally invasive tooth extraction (avoiding excessive buccolingual or mesiodistal movements during the extraction process to prevent damage to the alveolar bone walls) and ARP without primary wound closure using deproteinized bovine bone mineral (Bio-Oss®, Geistlich Pharma AG, Wolhusen, Switzerland), absorbable collagen membrane((Bio-Gide®, Geistlich Pharma AG, Wolhusen, Switzerland) and collagen sponge dressing(BeiLing®, Beijing HuiYouGuanHua Biology Technology Co., Ltd., Beijing, China) [41].

Records of patients who met the exclusion criteria were excluded: a) pregnancy or lactation; b) affected by systemic disease or medication that influences bone metabolism during the perioperative period; c) had a history of head and neck radiotherapy; d) smoked more than ten cigarettes per day; e) treated with tooth extraction due to caries, endodontic failure or fractured teeth; f) lack of Cone beam computed tomography (CBCT) taken before extraction or at least four months after the surgery.

### CBCT measurement at baseline

CBCT images were acquired with a three-dimensional x-ray unit (NewTom VG; Aperio Services, Italy) at a 0.125–0.300 mm resolution. At least two CBCT scans were needed, one taken before extraction (CBCT1) and one taken more than four months after surgery (CBCT 2).

The reference dots and vertical/horizontal lines were determined [10, 23]. In CBCT 1, residual height measurements were made at the central buccal (BH) and lingual/palatal (LH/PH) crests in the coronal planes and at points in the center of the mesial (MH) and distal (DH) margins of the sockets in the sagittal planes. In addition, the height at the center of the sockets (CH) was measured between the most coronal points of the maxillary sinus floor or mandibular canal and the most apical points of the extraction sockets.

**Fig. 1:**
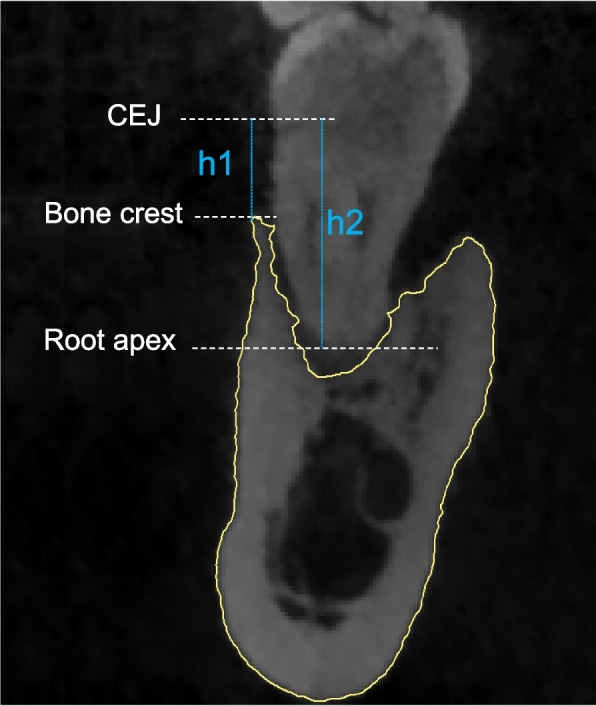
Schematic diagram for measurement of baseline bone loss (Baseline bone loss = h1/h2)

The baseline bone loss ratio was assessed in CBCT 1 at the central buccal and lingual/palatal crests by calculating the ratio of the distance between the cementoenamel junction (CEJ) and the alveolar crest to the distance between the CEJ and the root apex (Fig. 1). The degree of bone loss was defined according to the ratio: Grade I: bone loss ratio less than 50%; Grade II: bone loss ratio 50% ~ 75%; Grade III: bone loss ratio more than 75% [21].

Classification of baseline bone defects (Determined by buccal and lingual/palatal bone wall defect):

· Type I (Both buccal and lingual/palatal walls exist): buccal and lingual walls both were Grade I or Grade II;
· Type II (Buccal or lingual/palatal wall loss): either buccal or lingual/palatal wall was Grade III;
· Type III (Both buccal and lingual/palatal walls loss): Both buccal and lingual walls were Grade III.

### Virtual implant placement

**Fig. 2:**
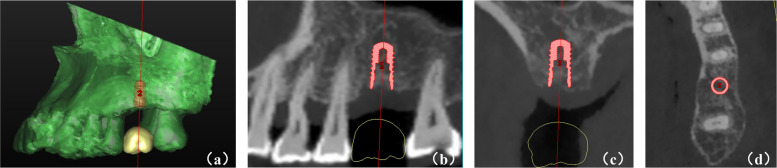
Virtual implant placement procedure. (**a**) Virtual crown setup; (**b**) Sagittal view after virtual implant placement; (**c**) Coronal view after virtual implant placement; (**d**) Cross-sectional view after virtual implant placement

The DICOM data of CBCT 2 were imported and reconstructed in Simplant Pro 17.0 (Dentsply Sirona Inc., PA, York, USA). Then, the software formed a standard virtual crown, adjusted according to adjacent and contralateral teeth, and placed in the appropriate position (Fig. 2). All sites received virtual implants with 4.1- or 4.8-mm platform diameters, and 8- or 10-mm implant lengths (Straumann® BL, Basel, Switzerland). The same examiner (HZ) positioned prosthetically driven virtual implants at included sites. The proper position was adjusted based on individual patient occlusion and adequate distance to adjacent teeth/implants in sagittal, coronal, and axial planes [30, 42, 43].

### Determining outcomes for virtual implant

**Fig. 3:**
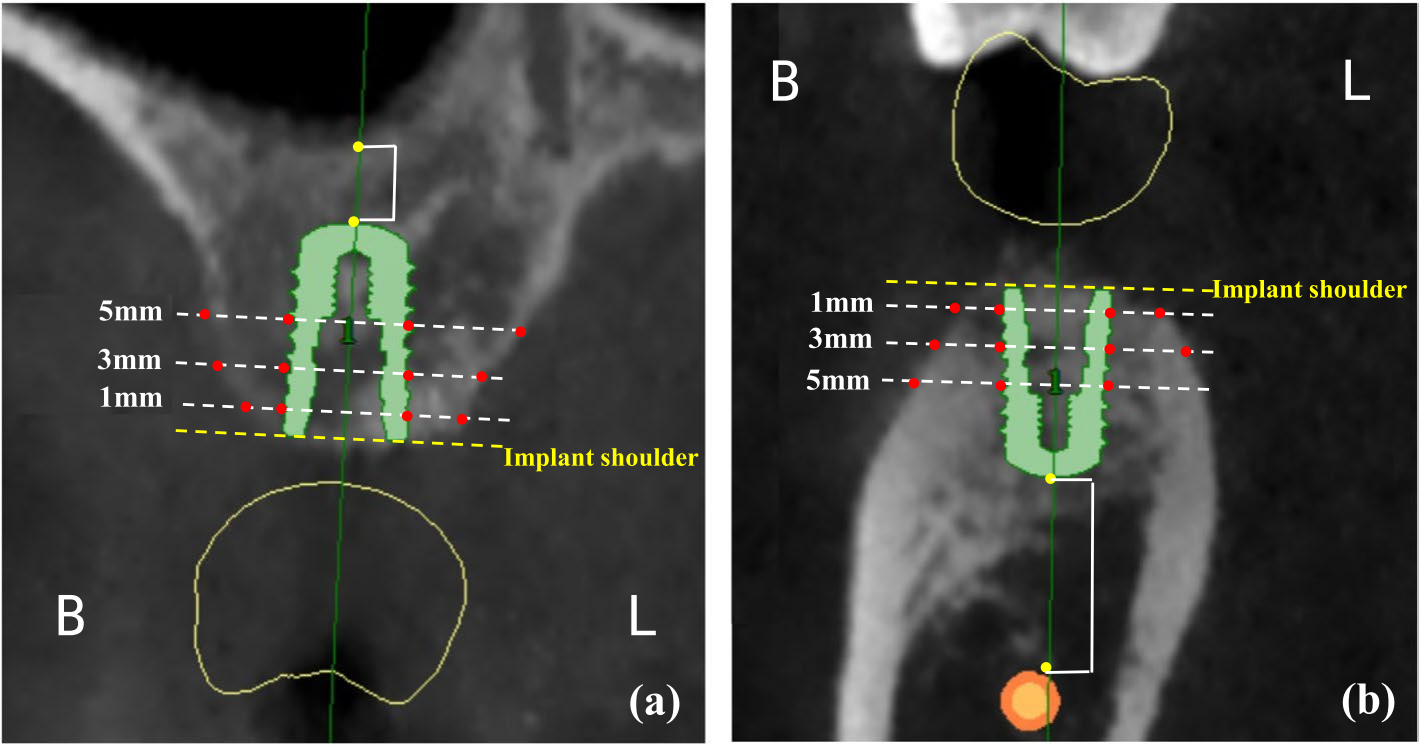
Residual bone height and width measurements following virtual implant placement. (**a**) Schematic diagram of maxillary measurements; (**b**) Schematic diagram of mandibular measurements

Cross sections of CBCT images at the center of virtual implants (Along the long axis of the implant and perpendicular to the arch form) were selected for assessment (Fig. 3). The following data were measured [30, 43]:

· Residual buccal and lingual/palatal bone thickness at 1, 3, and 5 mm apically from the implant shoulder. When the virtual implant was completely in bone and measurements of all sites were greater than 1.5 mm, it is defined as no simultaneous guided bone regeneration (GBR) procedure required at implant surgery. However, if one or more sites had deficient thickness (< 1.5 mm), it was determined that simultaneous GBR should be performed during implant placement. If the present ridge volume was inadequate for primary stability, staged GBR should be performed before implant placement.
· Distance from the initial floor of the maxillary sinus to the most apical part of implants: If the most apical part of the implant entered the maxillary sinus, osteotome sinus floor elevation (OSFE) was needed for distances of 3 mm or less, and lateral window sinus elevation (LWSE) should be performed for distances of more than 3 mm.
· Distance between the most apical part of the implant and the superior wall of the mandibular canal: ≥ 2 mm – virtual implants could keep a safety distance from the nerve canal; < 2 mm – there was no safety distance after virtual implants placement; the most apical part of implants offended the mandibular canals—implant placement was impossible or extensive vertical augmentation was required.

Treatment planning for implant placement based on measurements above:

· Standard procedure: implant placement without any bone augmentation.
· Simultaneous augmentation: simultaneous GBR or OSFE should be performed during implant placement.
· Staged augmentation: staged GBR or LWSE should be performed before implant placement.
· No possible or extensive vertical augmentation: the residual bone height above the mandibular canal is extremely insufficient.

### Results of clinical implant therapy

For sites treated with clinical implant therapy, the treatment modalities for implant placement were planned based on the residual bone width and height [44–46]. Both CBCT measurements and actual clinical observations were considered. The various implant treatment modalities were obtained from the medical records.

· Standard procedure: implant placement without any bone augmentation.
· Simultaneous augmentation: implant therapy with simultaneous GBR and/or OSFE.
· Staged augmentation: implant therapy with staged GBR and/or LWSE.

### Sample size calculation

The sample size was calculated using the Power Analysis and Sample Size (PASS) (version 15.0, NCSS, LLC, East Kaysville, Utah, USA) based on a two-sided alpha of 0.05. According to previous literature [41], the standard deviation of ridge height change in the middle of socket was 1.1 mm and the tolerance was 0.2 mm. After the calculation, the minimal sample size was 117 extraction sites for this study. The subjects included in this study met the sample size requirement.

### Data analysis

Statistical analysis was performed using Statistical Package for Social Sciences, version 26.0 (IBM SPSS, Chicago, IL, USA). A Shapiro–Wilk test was applied to test for the normal distribution of each continuous variable. Data that fit the normal distribution were reported as mean ± standard deviations. Other continuous data were described by median and interquartile range, and discrete variables were presented by frequency and percentage. Independent t-tests and Paired t-tests were performed to compare two groups of continuous variables. A non-parametric Mann–Whitney U test and Wilcoxon sign rank test were used to compare parameters that were not normally distributed, and the chi-square test was performed for discrete variables. A multinomial logistic regression analysis was performed to analyze the factors influencing implant treatment planning.

## Results

### Participants

A total of 313 teeth treated with extraction and ridge preservation that WH and TX performed at Peking University School and Hospital of Stomatology (PKUSS) were selected by chart review from January 2015 to July 2021. Eighty-one teeth were excluded due to inconsistency with inclusion and exclusion criteria. Another 41 teeth were excluded due to incomplete radiographic records (Figure S1). All included cases did not experience complications such as membrane exposure or postoperative infection after the surgery.

### Examiner reliability analysis

The same examiner (HZ) conducted all virtual implant placements and measurements. The examiner repeated CBCT measurements and virtual implant planning on 20 cases (10 from the maxilla and 10 from the mandible) two weeks apart. Substantial agreement was found between each repeated measurement. The intra-class correlations (ICCs) were 0.883 to 0.956 for CBCT measurements and 0.889 to 0.998 for indicators in virtual implant placement.

### Descriptive data

Finally, 143 subjects with 191 molars receiving minimal invasive extraction and ARP without primary closure were eligible. The characteristics are presented in TABLE S1. The baseline measurements of residual ridge height/width and differences between mandibular and maxillary sites are shown in TABLE S2. The measurements of residual ridge height were significantly higher in the mandible than in the maxilla.

### Main result

#### Results of virtual implant planning

Among all the included cases, the shortest healing time after ARP before virtual implant placement was 4 months, and the longest was 21.8 months, with an average healing time of 7.7 ± 2.9 months.

**Fig. 4:**
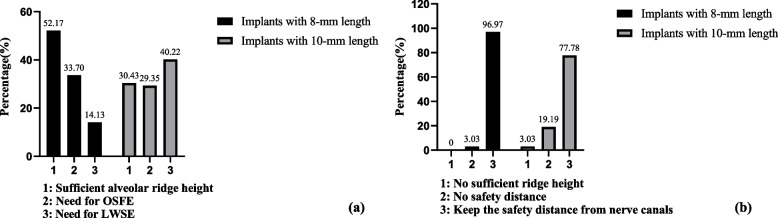
Need for sinus floor elevation in maxillary sites and classification of distances between the most apical part of the implant and the superior wall of the nerve canal in mandibular sites (placing implants with 8-/10-mm length). (**a**) Distribution of different implant treatment protocols in maxillary sites; (**b**) Distribution of different implant treatment protocols in mandibular sites. OSFE: Osteotome sinus floor elevation; LWSE: lateral window sinus elevation

Figure 4 presents the need for sinus floor elevation in maxillary sites and the classification of distances between the most apical part of implants and nerve canals in mandibular sites, respectively. When 8-mm implants were used in maxillary sites, 53.17% had sufficient ridge height. OSFE may be required in most sites needing sinus elevation (70.46%). When 10-mm implants were placed, LWSE procedure might be needed in 40.22% of the sites, and the complexity of implant surgery was likely to increase. All the mandibular sites had sufficient ridge height for receiving 8 mm implants, and most virtual implants (96.97%) could keep a safe distance from mandibular canals. However, 3.03% of the sites had insufficient height for 10-mm implants. Only 77.78% of the 10-mm virtual implants could keep a safe distance.

**Fig. 5:**
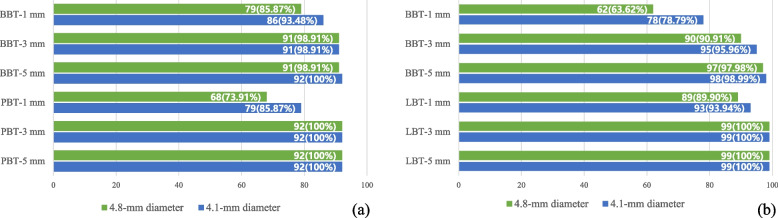
Percentage of cases with residual buccal/palatal bone thickness > 1.5 mm at 1-, 3- and 5- mm apically from the implant shoulder in (**a**) maxilla and (**b**) mandible (implant with 4.1-/4.8-mm diameter). BBT-1 mm: Residual buccal bone thickness at 1 mm apically from implant shoulder. BBT-3 mm: Residual buccal bone thickness at 3 mm apically from implant shoulder. BBT-5 mm: Residual buccal bone thickness at 5 mm apically from implant shoulder. PBT/LBT-1 mm: Residual palatal/lingual bone thickness at 1 mm apically from implant shoulder. PBT/LBT-3 mm: Residual palatal/lingual bone thickness at 3 mm apically from implant shoulder PBT/LBT-5 mm: Residual palatal/lingual bone thickness at 5 mm apically from implant shoulder

The percentage of cases with adequate residual buccal and lingual/palatal bone thickness (> 1.5 mm) at 1-, 3- and 5- mm apically from the implant shoulder in the maxilla and mandible is shown in Fig. 5.

Whether implants with 4.1- or 4.8-mm diameter were selected, adequate buccal and lingual/palatal bone thickness might be left at 3 mm and 5 mm apically from the implant shoulder in most cases. In the maxilla, the proportion of having inadequate palatal residual bone thickness at 1 mm apically from the implant shoulder was higher than that at the buccal side, and the results were opposite in mandibular sites.

#### Factors influencing virtual implant placement outcomes

A multinomial logistic regression analysis was performed to associate gender, age, location (maxilla or mandible), tooth type (1st or 2nd molar), classification of baseline bone defect (Type I, Type II or Type III), baseline bone loss ratio of mesial and distal crests, and the presence of the adjacent teeth (in mesial and distal side, YES/NO) with the implant placement planning (standard procedure, simultaneous augmentation and staged augmentation). Whether an implant (8 mm) with 4.1 mm or 4.8 mm diameter was placed, the most significant factors were the location (*p* < 0.001 & *p* < 0.001) and classification of baseline buccal and lingual/palatal bone wall defects (*p* < 0.01 & *p* < 0.001).

The outcomes for virtual implant planning are detailed in Table 1. The results suggest that all included sites are available for prosthetically guided virtual implant therapy. The proportion of standard implant placement procedures was higher in the mandible than the maxilla, regardless of whether a 4.1 × 8 mm or a 4.8 × 8 mm implant was used. It was observed that all cases requiring staged augmentation before implant placement were maxillary sites needing LWSE. Notably, none of the virtual implant sites were categorized as "staged augmentation" in cases with Type I baseline bone defects. It was found that cases with Type II baseline bone defects had the highest proportion of being categorized as "simultaneous augmentation".

**Table 1:** The proportion of outcomes for virtual implant placement

			**Standard procedure**	**Simultaneous augmentation**	**Staged augmentation**	***P***
4.1 × 8mm Implants	Location	Maxilla	41 (44.57%)	38 (41.30%)	13 (14.13%)	< 0.001*
		Mandible	72 (72.73%)	27 (27.23%)	0	
	Classification of baseline bone defect	Type I	61 (71.76%)	24 (28.24%)	0	< 0.001*
		Type II	37 (50.00%)	33 (44.59%)	4 (5.41%)	
		Type III	15 (46.88%)	8 (25.00%)	9 (28.12%)	
	Total		113 (59.16%)	65 (34.03%)	13 (6.81%)	191
4.8 × 8mm Implants	Location	Maxilla	33 (35.87%)	46 (50.00%)	13(14.13%)	< 0.001*
		Mandible	54 (54.55%)	45 (45.45%)	0	
	Classification of baseline bone defect	Type I	52 (61.18%)	33 (38.82%)	0	< 0.001*
		Type II	24 (32.43%)	46 (62.16%)	4 (5.41%)	
		Type III	11 (34.38%)	12 (37.50%)	9 (28.12%)	
	Total		87 (45.55%)	91 (47.64%)	13 (6.81%)	191

Classification of baseline bone defects (Determined by buccal and lingual/palatal bone wall defect):Type I-Both buccal and lingual/palatal walls exist; Type II-Buccal or lingual/palatal wall loss; Type III-Both buccal and lingual/palatal walls loss

Data in brackets present the percentage of this value in total number of the same row

*P* values came from Pearson chi square test

^*^Significant result, *P*-value <.05

#### Results of clinical implant therapy

Among all included sites, 159 received clinical implant therapy (Table 2). Implants with 4.1 mm or 4.8 mm diameter and a regular length of ≥ 8 mm (Straumann® BL, Basel, Switzerland) were placed in these sites. Of these cases, 121 sites (76.10%) received implant therapy without additional bone augmentation, and five sites (3.15%) received staged bone augmentation before implant therapy. In the maxilla, 36.12% of the sites received simultaneous bone augmentation during implant therapy (OSFE), and 6.94% received staged bone augmentation (LWSE) before implant therapy. Most sites (91.95%) in the mandible received implant therapy without bone augmentation; none received staged bone augmentation. In cases with Type I classification of bone wall defects, none of the sites received staged bone augmentation. There were no significant differences in the distribution of outcomes for clinical implant placement with different classifications of baseline bone defect (*P* = 0.104).

**Table 2:** Outcomes for clinical implant therapy

		**Standard procedure**	**Simultaneous augmentation**	**Staged augmentation**	***P***
Location	Maxilla	41 (56.94%)	26 (36.12%)	5 (6.94%)	< 0.001*
	Mandible	80 (91.95%)	7 (8.05%)	0	
Classification of baseline bone defect	Type I	59 (83.10%)	12 (16.90%)	0	0.104
	Type II	44 (67.69%)	17 (26.15%)	4 (6.15%)	
	Type III	18 (78.26%)	4 (17.39%)	1 (4.35%)	
Total		121 (76.10%)	33 (20.75%)	5 (3.15%)	159

Note: Among all included sites, 159 were treated with dental implant therapy

Classification of baseline bone defects (Determined by buccal and lingual/palatal bone wall defect): Type I-Both buccal and lingual/palatal walls exist; Type II-Buccal or lingual/palatal wall loss; Type III-Both buccal and lingual/palatal walls loss

Data in brackets present the percentage of this value in total number of the same row

*P* values came from Pearson chi square test and Fisher’s exact test

^*^Significant result, *P*-value <.05

## Discussion

The present investigation evaluated the possibility and need for bone augmentation in virtual implant planning and clinical implant therapy following ARP in periodontally compromised molar extraction sites.

Cha et al. and Wei et al. reported the treatment modalities for implant placement following ARP and natural healing in actual cases [20, 26]. According to Cha and colleagues, approximately 7.1% of the ARP sites received lateral sinus augmentation procedures, while the proportion was 28.6% in the natural healing group. Additionally, while no cases allowed standard implant procedure in natural healing sites, 42.9% of ARP cases permitted “implant placement without any additional sinus augmentation” [26]. Wei and colleagues found that simple implant surgery without sinus augmentation could be performed in 83.3% of the subjects in the ARP group, and 50% in the natural healing group needed sinus augmentation [20]. The results of the present investigation align with Cha and colleagues’ study; sinus floor elevation might be necessary in 47.83% of the sites in the maxilla when considering 8 mm-length implants. In maxillary cases receiving clinical implant therapy, 56.94% underwent simple implant procedures, and 6.94% needed staged bone augmentation before implant therapy. Abellán et al. assessed the clinical need for horizontal ridge augmentation and sinus elevation during implant placement after the ARP procedure using two different biomaterials. None of these cases needed the lateral approach, 55.56% of the sites received transcrestal sinus elevation, and only one case required further horizontal ridge augmentation during implant surgery [27]. In the present results of virtual implant planning, 40.80% of the ARP sites might require horizontal bone augmentation. In the mandibular sites that received clinical implant therapy, 8.05% of these sites performed simultaneous horizontal bone augmentation during implant therapy. These proportions are higher than the previous research. However, the sample size of Abellán’s study was relatively small. In research based on data obtained from implant placement in actual cases, it cannot be ignored that the deviation in implant surgery and implant preferences of different surgeons (different implant systems, variable implant diameter and length, various criteria for augmentation procedures) do exist and make it difficult to compare the results [47]. Standardized virtual implant placement procedures may enhance cross-study comparability.

Fok and colleagues placed virtual implants in maxillary and mandibular first molar extraction sites, which had natural healing due to severe periodontitis. They assessed the proportion of standard implant placement and the need for simultaneous or staged bone augmentation [30]. The percentage of sites requiring LWSE in the maxilla was 41.2% (when considering 4.1 × 8 mm or 4.8 × 8 mm implants), and the proportions of standard implant procedures in the mandible are 60.9% for 4.1 × 8 mm implants and 43.5% for 4.8 × 8 mm implants. The present study performed comparable result evaluations and selection for virtual implants. The results showed that 14.1% of the selected sites in the maxilla may require LWSE when choosing 4.1/4.8 × 8 mm implants, which is much lower than the previous study [30]. Conversely, the present study’s proportion of standard implant procedure in mandible was 72.7% (for 4.1 × 8 mm implants) and 54.5% (for 4.8 × 8 mm implants), which is higher than that in the previous study [30]. In another study, An and colleagues evaluated unassisted bone healing within infected extraction sockets following prosthetically driven implant placement [48]. This study showed that most implants (44 in 48 sites) could be virtually placed in the center of edentulous sites without surface exposure. In that study, implants with 4–5 mm diameter and 8–12 mm length were chosen, and subsequent measurements, such as residual bone thickness around implants, were not performed. Koutouzis et al. assessed the necessity for additional regenerative procedures following the healing of compromised and non-compromised extraction sockets with ARP by virtual implant placement [49]. They found that 36.8% of the implants exposed surfaces for compromised molars (implants with a diameter of 4.8 mm were selected in molar sites). The assessment of the need for additional augmentation procedures is stricter in the present study, and the outcomes are similar to those of Koutouzis & Lipton. The outcomes of virtual implant placement in periodontal compromised molar sites following ARP and unassisted socket healing should be compared in further studies.

In the present study, according to the logistic regression analysis, the potential influencing factors for treatment planning of virtual implant placement were the location and classification of baseline buccal and lingual/palatal bone wall defects. When considering the impact of location, we found that the proportion of simple implant placement without bone augmentation was higher in the mandible than in the maxilla, and all the cases that needed staged augmentation were sites requiring LWSE in the maxilla. Fok et al. demonstrated a similar trend [30]. Few studies directly compared ridge dimentional changes and implant outcomes between the maxilla and mandible following ARP. Leblebicioglu et al. [36] found no significant difference in bone width changes between jaws after ARP. However, maxillary sites exhibited a height loss of 0.2 ± 0.3 mm, while mandibular sites showed a height gain of 1.0 ± 0.3 mm, with no significant differences. Regarding implant outcomes, Lee and colleagues reported that the maxillary posterior region was also a potential influencing factor in vertical bone augmentation (OR = 0.25, *P* = 0.015), where the sinus floor elevation procedure was included in vertical bone augmentation [28]. The discrepancy in outcomes of different locations in the present investigation may be due to the significantly higher baseline measurements of residual ridge height in the mandible than in the maxilla (Table S2).

In this study, we provided results of virtual implant planning and clinical implant therapy. The results showed a lower percentage of the need for additional bone augmentation during the clinical implant therapy, and the possible reasons are as follows. Firstly, some patients who required complicated bone augmentation did not receive implant therapy and chose alternative prosthetic modalities. Secondly, in some sites, bone augmentations were circumvented by adjusting the position and angulation of the implants within the appropriate range. In clinical scenarios, no additional bone augmentation is required when the peri-implant bone thickness reaches more than 1 mm. Furthermore, there were some inaccuracies in the assessment of bone volume using CBCT. Therefore, evaluating the results of both virtual implant planning and clinical implant procedures is necessary.

This study is a retrospective observational study subject to certain limitations. The limitations include data quality and completeness issues, challenges in establishing causality due to confounding factors and temporal uncertainties, and potential selection bias. These factors necessitate cautious interpretation of the findings. The main limitation of this investigation is the lack of a control group. Consequently, the evaluation of the benefits of ARP over unassisted socket healing in periodontally compromised molars was not achieved. However, the present investigation generated all the data and medical records in routine clinical procedures and performed statistical analysis on many subjects. To the authors' knowledge, this investigation is one of the few studies that have provided the outcomes of virtual implant placement and clinical implant procedures in compromised molar sockets receiving ARP. In summary, the key findings of this study are as follows:

· Through virtual implant planning and actual clinical outcome evaluation, this study demonstrated that prosthetically guided dental implants are viable in periodontally compromised molars following ARP, with only a few sites requiring complicated bone augmentation procedures.
· Location (maxillary/mandible) and buccal and lingual/palatal bone defects are the influencing factors for implant treatment planning. The proportion of standard implant placement procedures was higher in the mandible than the maxilla, and all sites requiring staged augmentation were maxillary sites. With the increase in severity of baseline bone defect, the proportion and complexity of cases requiring bone augmentation during implant treatment also increase.

## Supplementary Information

Supplementary Material 1. Table S1: Characteristics of study subjects.

Supplementary Material 2. Table S2: Baseline measurements and differences between maxilla and mandible.

Supplementary Material 3. Figure S1: The flowchart of this study.

## Data Availability

The data supporting this study's findings are available from the corresponding author upon reasonable request.
