# Supplementary material for: Virtual and clinical implant placement after ridge preservation in periodontally compromised molars: retrospective study
Source: BMC Oral Health. 2026 Feb 18;26:526. doi: 10.1186/s12903-026-07921-7 (PMC13020102; doi:10.1186/s12903-026-07921-7)
Supplement: Supplementary file 1 — Supplementary Material 1. Table S1: Characteristics of study subjects. [file 12903_2026_7921_MOESM1_ESM.docx]

**TABLE S1 Characteristics of study subjects (tooth-level)**

| **Subjects’ demographics** | |  |
| --- | --- | --- |
| **Age at surgery (years)** | | 49.63 ± 9.29 |
| **Healing period (days)** | | 229.50 ± 86.50 |
| **Gender** | Female | 67 (35.1%) |
|  | Male | 124 (64.9%) |
| **Tooth type** | Maxillary 1^st^ molar | 57 (29.8%) |
|  | Maxillary 2^nd^ molar | 35 (18.3%) |
|  | Mandibular 1^st^ molar | 38 (19.9%) |
|  | Mandibular 2^nd^ molar | 61 (31.9%) |
| **Presence of adjacent teeth in mesial side** | YES | 154 (80.6) |
|  | NO | 37(19.4%) |
| **Presence of adjacent teeth in distal side** | YES | 81 (42.4%) |
|  | NO | 110 (57.6%) |
| **Classification of baseline buccal and lingual bone defect** | Type I | 85 (44.5%) |
|  | Type II | 74 (38.7%) |
|  | Type III | 32 (16.8%) |
| **Baseline ridge height** | Buccal | 5.59 ± 2.67 |
|  | Lingual/Palatal | 5.83 ± 2.62 |
|  | Mesial | 7.53 ± 2.81 |
|  | Distal | 6.75 ± 2.86 |
|  | Central | 4.92 (4.82) |
