# Supplementary material for: Virtual and clinical implant placement after ridge preservation in periodontally compromised molars: retrospective study
Source: BMC Oral Health. 2026 Feb 18;26:526. doi: 10.1186/s12903-026-07921-7 (PMC13020102; doi:10.1186/s12903-026-07921-7)
Supplement: Supplementary file 2 — Supplementary Material 2. Table S2: Baseline measurements and differences between maxilla and mandible. [file 12903_2026_7921_MOESM2_ESM.docx]

**Table S2 Baseline measurements and differences between maxilla and mandible（mm）**

|  | **Indicators** | **Location** | **Mean ±standard deviation /**  **Median (interquartile range)** | **P** |
| --- | --- | --- | --- | --- |
| **Ridge height** | CH | Maxilla | 3.30 (4.63) | ＜0.001* |
|  |  | Mandible | 6.22 (4.29) |  |
|  | BH | Maxilla | 5.13 ± 2.83 | 0.023 |
|  |  | Mandible | 6.01 ± 2.45 |  |
|  | LH/PH | Maxilla | 4.70 ± 2.45 | ＜0.001* |
|  |  | Mandible | 6.88 ± 2.32 |  |
|  | MH | Maxilla | 6.81 ± 2.70 | ＜0.001* |
|  |  | Mandible | 8.21 ± 2.76 |  |
|  | DH | Maxilla | 6.06 ± 2.44 | 0.001* |
|  |  | Mandible | 7.39 ± 3.08 |  |

Note: P values came from Independent t-test and non-parametric Mann-Whitney U test

*Significant result, p-value < .05

CH: ridge height in the center of the sockets; BH: ridge height at central buccal crest; LH/PH: ridge height at lingual/palatal crest; MH: ridge height at point in the center of mesial margin of socket; DH: ridge height at point in the center of distal margin of socket.
