# Supplementary material for: Virtual and clinical implant placement after ridge preservation in periodontally compromised molars: retrospective study
Source: BMC Oral Health. 2026 Feb 18;26:526. doi: 10.1186/s12903-026-07921-7 (PMC13020102; doi:10.1186/s12903-026-07921-7)
Supplement: Supplementary file 3 — Supplementary Material 3. Figure S1: The flowchart of this study. [file 12903_2026_7921_MOESM3_ESM.pdf]

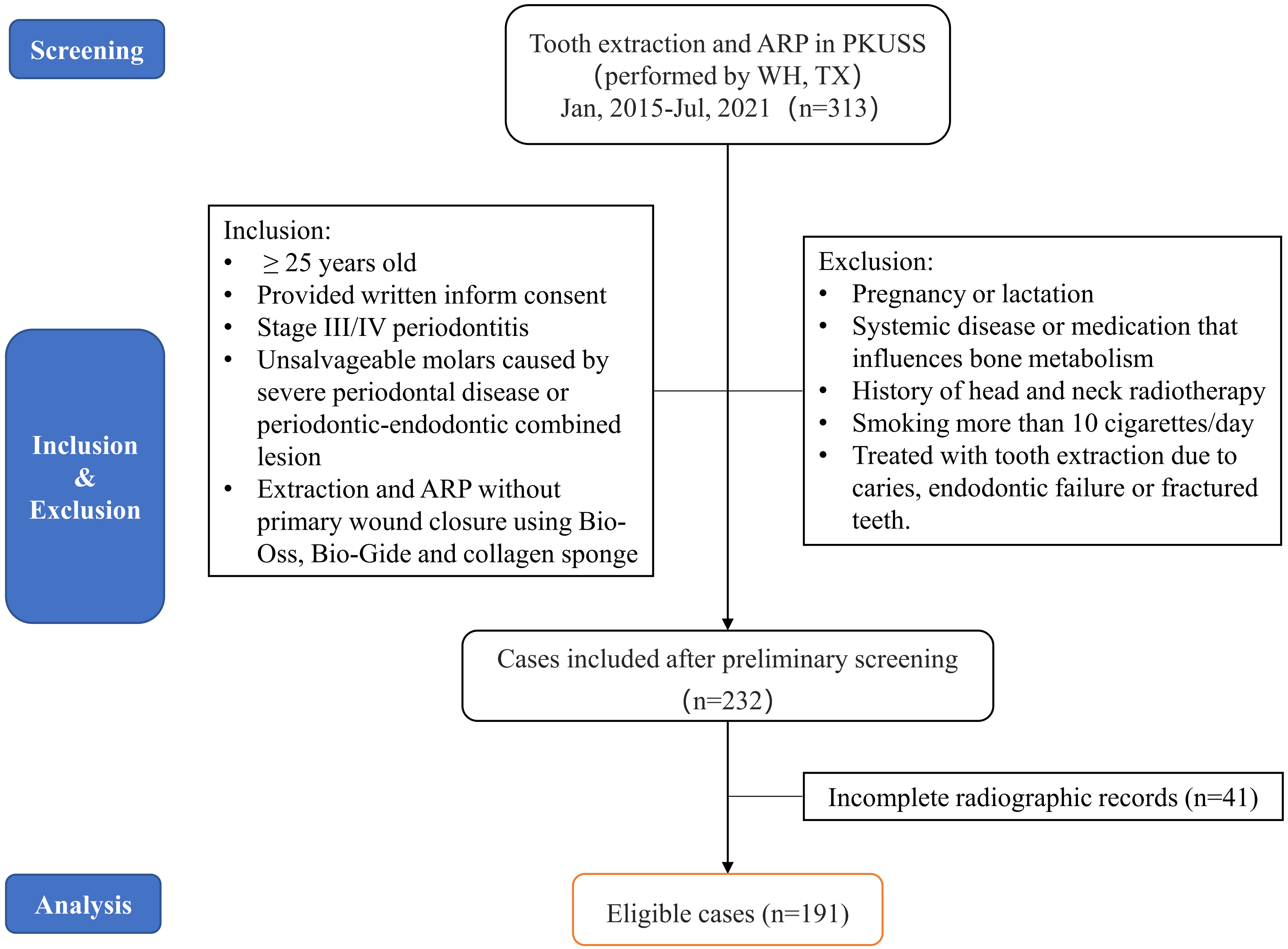
Screening
Tooth extraction and ARP in PKUSS (performed by WH, TX) Jan, 2015-Jul, 2021 (n=313)
Inclusion & Exclusion
Inclusion:
• ≥ 25 years old
• Provided written inform consent
• Stage III/IV periodontitis
• Unsalvageable molars caused by severe periodontal disease or periodontic-endodontic combined lesion
• Extraction and ARP without primary wound closure using Bio-Oss, Bio-Gide and collagen sponge
Exclusion:
• Pregnancy or lactation
• Systemic disease or medication that influences bone metabolism
• History of head and neck radiotherapy
• Smoking more than 10 cigarettes/day
• Treated with tooth extraction due to caries, endodontic failure or fractured teeth.
Cases included after preliminary screening (n=232)
Incomplete radiographic records (n=41)
Analysis
Eligible cases (n=191)
